# Characteristics and prognosis of gastric cancer in young patients

**DOI:** 10.3892/or.2013.2467

**Published:** 2013-05-15

**Authors:** TARO ISOBE, KOUSUKE HASHIMOTO, JUNYA KIZAKI, MOTOSHI MIYAGI, KEISHIRO AOYAGI, KIKUO KOUFUJI, KAZUO SHIROUZU

**Affiliations:** Department of Surgery, Kurume University School of Medicine, Fukuoka, Japan

**Keywords:** gastric cancer, prognosis, young, elderly

## Abstract

The clinicopathological features of gastric cancer (GC) differ between younger and older patients, and it is thought that younger patients have a worse prognosis than older patients due to delayed diagnosis and more aggressive tumor behavior. These characteristics, however, remain controversial. A total of 3,818 patients with pathologically confirmed primary gastric adenocarcinoma were treated at our institution. We analyzed the difference in demographic and clinicopathological characteristics between 169 young [≤40 years of age, younger group (YG)] and 3,649 older [>40 years of age, older group (OG)] GC patients. There was a significantly higher proportion of females in the YG compared with the OG (53.3 and 31.0%, respectively; P<0.0001). The 5-year overall survival of the YG was significantly lower compared to that of the OG (59.7 and 65.9%, respectively; P=0.049). However, YG patients with curative resection had a similar 5-year survival rate to OG patients with curative resection (88.0 and 85.8%, respectively; P=0.547). Female patients in the YG showed a significantly lower survival rate than males in the YG (44.3 and 73.1%, respectively; P=0.0002). Multivariate analyses revealed that macroscopic type, depth of invasion, peritoneal metastasis, distant metastasis and curative resection were independent prognostic factors for the YG with GC. Young GC patients who undergo curative resection do not have a worse prognosis than older patients. Early diagnosis is important in successfully carrying out a curative resection and offering a better prognosis, particularly in females.

## Introduction

Gastric cancer (GC) is one of the most common causes of cancer-related death. Every year, 1 million new cases of GC are diagnosed and 700,000 die of this disease worldwide (1,2). Most patients with GC are diagnosed with advanced GC and overall survival remains poor. GC is generally considered to be an age-related disease and although more than half of GC patients are ≥70 years of age, some studies have revealed that 2.0–8.0% of patients with GC are ≤40 years of age (3–5). Although the incidence of advanced GC has steadily decreased because of recent developments in medical screening, GC in young people remains a serious problem as routine screening in Japan does not include people <40 years of age. GC is difficult to diagnose in young people and is asymptomatic even in the advanced stages of the disease.

The prognosis for young patients remains controversial. Clinicopathological features of GC are reported to differ between younger and older patients and it is thought that the prognosis of the disease is worse for younger patients due to delayed diagnosis and more aggressive tumor behavior (6–9). However, other reports state that tumor staging and prognosis for younger patients is similar to older patients and is dependent upon whether or not the patient undergoes a curative resection (5,10,11). In order to clarify the prognosis of younger patients with GC, we analyzed the differences in demographic and clinicopathological characteristics between younger (≤40 years of age) and older (>40 years of age) GC patients.

## Materials and methods

From 1977 to 2006, a total of 3,818 patients with pathologically confirmed primary gastric adenocarcinoma were consulted and 3,563 underwent gastric resection at the Department of Surgery at Kurume University School of Medicine. Patients were monitored for at least 5 years after surgery every 1–3 months and examined by computed tomography (CT) scan, ultrasound and upper endoscopy at least once a year. Patients with diagnoses of squamous cell carcinoma, adenosquamous cell carcinoma, small cell carcinoma, carcinoid tumor, lymphoma or gastrointestinal stromal tumors were excluded.

**Figure 1:**
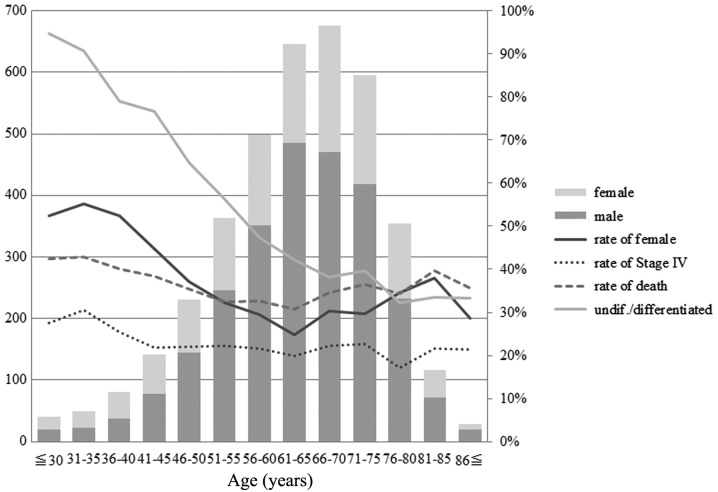
Distribution of gender frequency, undifferentiated cancer type, stage IV disease and 5-year survival were examined to define the appropriate age groups for comparison.

The distribution of gender frequency, undifferentiated cancer type, stage IV disease and survival at 5 years was examined to define appropriate age groups for comparison (Fig. 1). These demographic and clinicopathological features tended to be different between the patients aged 40 years or less and those aged over 40 years. Thus, we divided our population into 2 groups according to age with a cut-off of 40 years. The younger group (YG) was comprised of 169 patients (4.43%) ≤40 years of age while the older group (OG) was comprised of the remaining 3,649 patients >40 years of age. In the YG, 79 were male (2.07%) and 90 were female (2.36%), while in the OG 2,518 were male (66.0%) and 1,131 were female (29.6%). The median ages were 34.5±4.8 years (range, 20–40 years) for the YG and 64.5±10.0 years (range, 41–92) for the OG. The male-to-female ratios in the YG and OG were 1:1.14 and 1:0.45, respectively, with a significantly higher proportion of females in the YG compared with the OG (P<0.0001). Patient records retrospectively examined for gender, family history of GC, clinicopathological factors, surgical procedures and survival. The tumors were staged according to the guidelines of the Japanese Classification of Gastric Carcinoma (Japanese Gastric Cancer Association) (12).

Total or distal (subtotal) gastrectomy was performed according to the tumor size and location, status of resection margins and lymph node involvement. The standard procedure was a spleen- and pancreas-preserving D2 or D3 lymph node dissection. Surgery was considered curative when all resection margins were clear, there was no or minimal serosal invasion, nodal involvement was N2 or less, there was an absence of tumor invasion in the last lymph node resected barrier, and there was no evidence of spread to the liver, peritoneum, or ovaries at laparotomy. Sixty-nine (40.8%) of the YG patients and 1,180 (32.3%) of the OG patients were randomly assigned to various regimens of chemotherapy (P=0.021), including 5-fluorouracil, cisplatin, mitomycin C, and paclitaxel, among others. Nine (0.2%) patients in the OG and no patients in the YG received neoadjuvant chemotherapy (5-fluorouracil/cisplatin combination).

Clinical records were compared by either Fisher’s exact test or Pearson’s χ^2^ test, as appropriate. Survival rate was calculated by the Kaplan-Meier method, and univariate analyses used the log-rank test. Factors that were deemed of potential importance to the univariate analysis were included in the multivariate analysis using the Cox proportional hazard model. A P<0.05 was considered a statistically significant result. Data analysis was performed using the statistical program JMP^®^ 8 (SAS Institute, Cary, NC, USA).

## Results

The clinicopathological characteristics of the GC patients are compared in Table I. There was no statistically significant difference between the YG and OG regarding family history of GC (5.9 vs. 6.3%, P=0.851). The proportion of tumor lesions located in the middle third or involving the whole stomach was significantly higher in the YG than in the OG (41.4 vs. 28.7%, P=0.0004; 13.6 vs. 9.0%, P=0.044, respectively), while the occurrence of tumor lesions in the lower third of the stomach was significantly higher in the OG than that in the YG (23.7 vs. 36.8%, P=0.0005). There was no statistically significant difference among the proportion of esophageal or duodenal invasion or of the occurrence of gastric stump (P=0.556, P=0.312 and P=0.071, respectively). Regarding macroscopic lesion types, Borrmann type 4 (diffuse infiltrative) lesions were more common in the YG compared with the OG (23.7 vs. 9.5%, P<0.0001), while Borrmann type 0 (superficial), type 1 (mass), type 2 (ulcerative) lesions were more common in the OG compared with the YG (36.7 vs. 46.2%, P=0.016; 0 vs. 2.4%, P=0.042; 6.5 vs. 11.8%, P=0.035, respectively). With regard to histological type, significantly more patients in the YG had poorly differentiated adenocarcinoma and signet ring cell carcinoma (39.1 vs. 25.4%, P=0.0002; 44.4 vs. 16.4%, P<0.0001, respectively), while more patients in the OG had papillary adenocarcinoma and tubular adenocarcinoma (0 vs. 4.5%, P=0.008; 14.2 vs. 51.1%, P<0.0001, respectively). Depth of invasion, peritoneal metastasis and stage of disease status had a significantly greater incidence in the YG than in the OG (P=0.010, P=0.0014 and P=0.019, respectively). Both groups had similar distributions with respect to lymph node metastasis, the mean number of metastatic lymph nodes, hepatic metastasis and distant metastasis.

Surgical characteristics are summarized in Table II. In the YG, 152 patients (89.9%) had surgical resection while 3,411 (93.5%) of the OG patients had surgical resection (P=0.072); 112 (73.7%) YG patients and 2,728 (80.0%) OG patients had curative resection. The curative resection rate in the YG tended to be lower than that in the OG (P=0.059). The proportion of ‘open and closure’ in the YG was higher than that in the OG, due to the unresectable situation (P=0.037). The incidence of total gastrectomy and distal gastrectomy were similar in the YG and OG (P=0.138 and P=0.879, respectively). Proximal gastrectomy or partial resection (segmental or wedge gastrectomy), so-called reduction surgery, was frequently performed in the OG due to the comorbidities or general conditions present in this group (P=0.041 and P=0.005, respectively). There was a higher proportion of D2 and D3 lymphadenectomy in the YG compared with the OG (P=0.0015). Regarding the combined resection, pancreas tail, spleen, transverse colon and ovary were highly resected in the YG compared with the OG (P=0.0001, P=0.010, P<0.0001 and P<0.0001, respectively).

**Figure 2:**
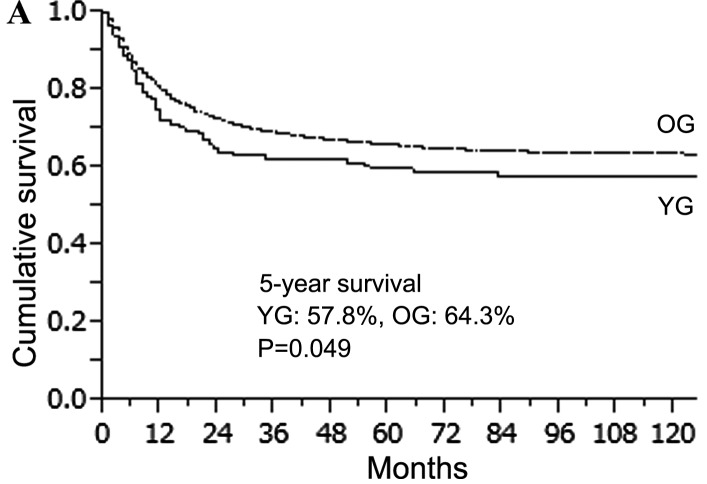

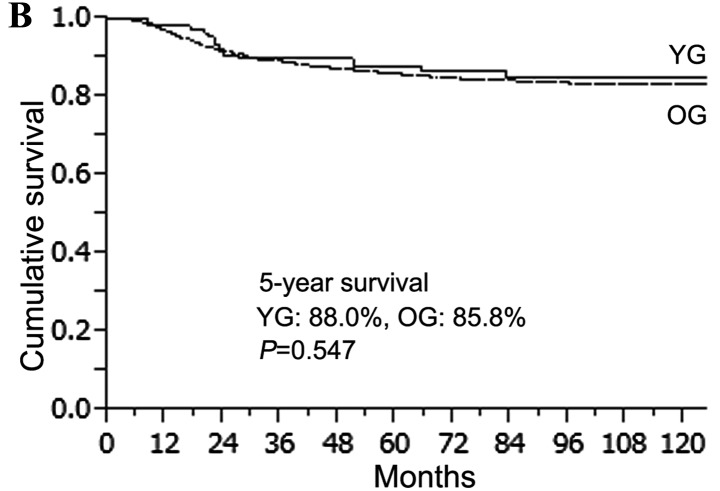
Survival curves according to age for the younger group (YG) and the older group (OG). (A) All patients and (B) patients with curative resection.

**Figure 3:**
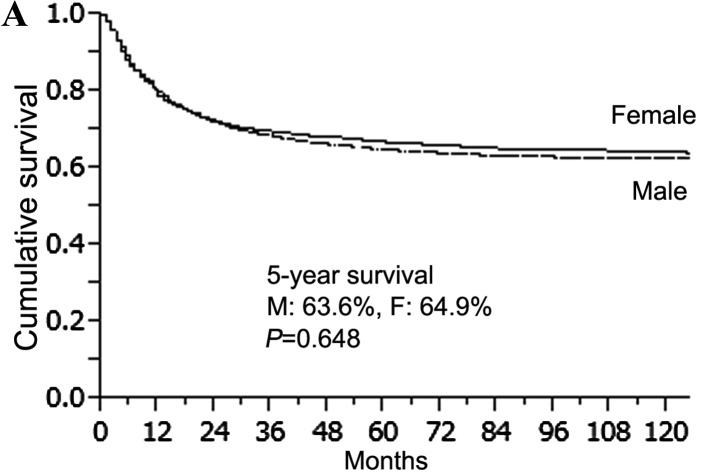

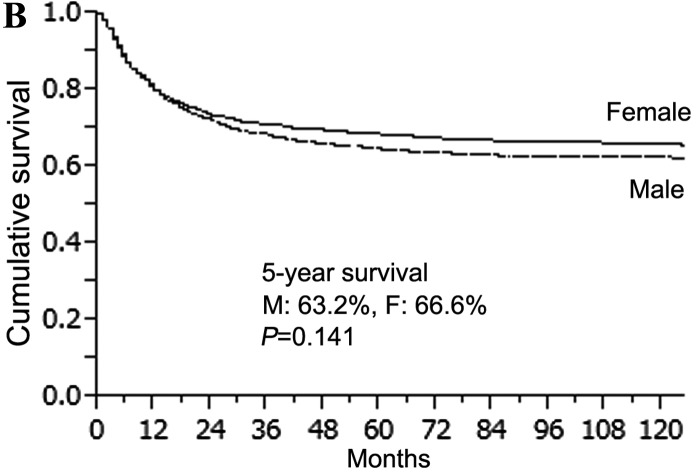

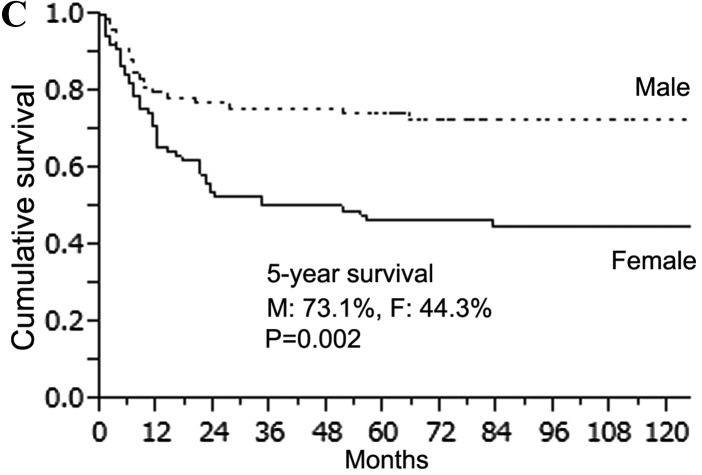
Survival curves according to gender. (A) All patients, (B) older group (OG) and (C) younger group (YG).

**Figure 4:**
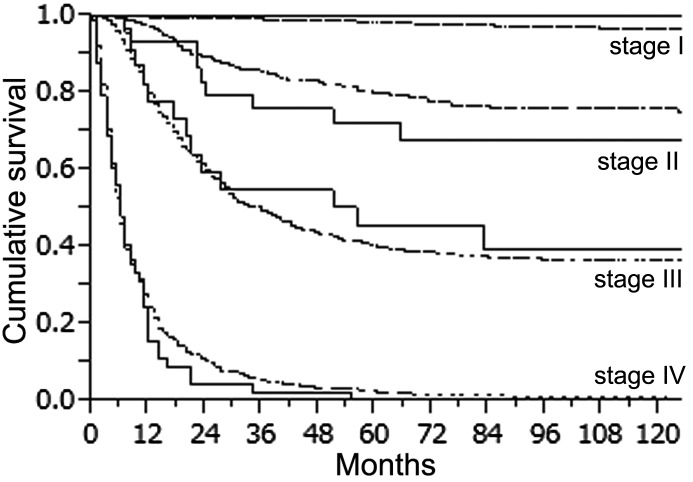
Survival curves according to stage for the younger group (YG, solid line) and the older group (OG, dashed line). The 5-year survival rate of the YG and OG at stage I (100 vs. 97.3%, P=0.181), stage II (68.6 vs. 76.7%, P=0.286) and stage III (36.6 vs. 37.4%, P=0.760). The 2-year survival rate of the YG and OG at stage IV was 4.4 vs. 10.4% (P=0.612).

**Figure 5:**
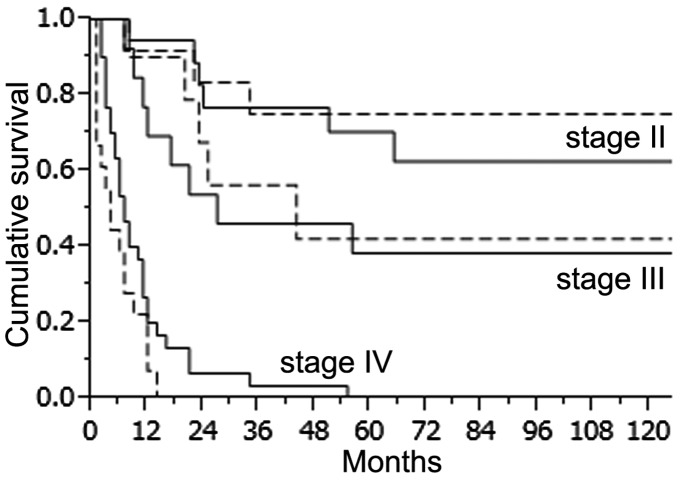
Survival curves for the younger group (YG) according to stage on patients treated with chemotherapy (CG, solid line) and patients not treated with chemotherapy (NCG, dashed line). The 5-year survival rate of the CG and NCG at stage II was 70.3 vs. 75.0% (P=0.646) and at stage III was 38.5 vs. 42.2% (P=0.568). The 2-year survival rate of the CG and NCG at stage IV was 6.7 vs. 0% (P=0.612).

The overall median follow-up was 65.1 months (range 0–256 months). The 5-year overall survival rate in the YG and OG was 57.8 and 64.3%, respectively (Fig. 2A). The OG survival rate was significantly higher than that of the YG (P=0.049). However, patients in the YG with curative resection had a similar 5-year survival rate to those in the OG with curative resection (88.0 vs. 85.8%, P=0.547) (Fig. 2B). When the 5-year survival rate was compared with gender, there was no significant difference in survival for all patients or those in the OG (male 63.6% vs. female 64.9%, P=0.648; male 63.2% vs. female 66.6%, P=0.141) (Fig. 3A and B). However, female patients in the YG showed a significantly lower survival rate than males in the YG (female 44.3% vs. male 73.1%, P=0.0002) (Fig. 3C). When survival was determined according to the stage of the disease, there was no statistically significant difference in survival rate for all stages between the 2 groups (Fig. 4). However, stage IV patients in the YG had a slightly worse outcome than the pacients in the OG. The 1-year survival rate in the YG and OG was 15.6 and 24.2%, respectively and the 2-year survival rate was 4.4 and 10.4%, respectively. We also compared survival in the YG determined according to the stage (II–IV) between patients treated with chemotherapy (CG) and those not treated with chemotherapy (NCG). The 5-year survival rate of CG and NCG patients were as follows: stage II (70.3 vs. 75.0%, P=0.646); stage III (38.5 vs. 42.2%, P=0.568). The 2-year survival rate of CG and NCG patients at stage IV was 6.7 vs. 0% (P=0.612). There was no significant difference in survival rate for all stages between the 2 groups (Fig. 5).

Analyses of the prognostic factors for the YG in GC are presented in Table III. Macroscopic type, depth of invasion, peritoneal metastasis, distant metastasis and curative resection emerged as independent prognostic factors (P=0.014, P=0.041, P=0.001, P=0.018 and P=0.021, respectively).

## Discussion

GC is usually a disease of the aged, with the mean patient age ranging between 50 and 70 years. It is thought that GC results from a combination of environmental factors and an accumulation of generalized and specific genetic alterations, consequently affecting primarily older patients after a long period of atrophic gastritis (13). Intestinal-type cancers develop as a result of chronic atrophic gastritis and subsequent intestinal metaplasia is primarily associated with chronic *Helicobacter pylori* infection (13,14). Younger patients have fewer years to develop intestinal metaplasia, which may partially explain why they disproportionately present with a higher proportion of diffuse cancers. The diffuse type of GC is common in young patients with genetic predisposition (presence of *CDH1*), one of the major factors involved in the development of GC (2,15,16). Although the underlying genetic events are not always known, they can involve *CDH1* germline mutations, which encode an aberrant form of E-cadherin, resulting in hereditary diffuse GC (17–19). In this study, 5.9% of the YG patients with GC had a positive family history of GC and there was no statistically significant difference compared with the OG (P=0.851). The proportion of tumor lesions located in the middle third and involving the whole stomach was significantly higher in the YG than in the OG, although the presence of tumor lesions in the lower third of the stomach was significantly higher in the OG than in the YG. Regarding macroscopic types, Borrmann type 4 lesions were more common in the YG, while Borrmann type 0–2 lesions were more common in the OG. With regard to histological type, we found that poorly differentiated adenocarcinoma and signet ring cell carcinoma were more common in the YG, while more patients in the OG had evidence of papillary adenocarcinoma and tubular adenocarcinoma. The macroscopic and histological results presented here were comparable to other reports (5,20,21).

In the present study, there was a higher proportion of D2 and D3 lymphadenectomy in the YG compared with the OG. Both groups had similar distributions with respect to lymph node metastasis and the mean number of metastatic lymph nodes. Furthermore, reduction surgeries were frequently performed in the OG. These results may be due to the comorbidities or general conditions in the OG. There were 186 patients ≥80 years of age in this study (D0, 34; D1, 110; D2, 42). In Japan, radical gastrectomy with extended lymphadenectomy (D2) is commonly employed for GC, as this procedure results in higher stage-stratified survival compared to the Western method (22). However, in elderly patients, limited operations are usually employed to reduce surgical stress (23). However, other studies found that postoperative survival was not significantly different following D1 or D2 gastrectomy in GC patients over 80 years of age (24,25).

Some studies report female predominance among young patients with GC (26–28) and indeed, we found a female-to-male ratio of 1.14:1 in young patients (0.45:1 in older patients). In this study, there was no significant difference in the 5-year overall survival regarding gender in all patients (male 63.6% vs. female 64.9%, P=0.648). However, females had a lower survival rate than males in the YG (female 44.3% vs. male 73.1%, P=0.0002). This predominance of females is considered by some to be due to hormonal factors, such as the harmful role of estrogens, as well as higher percentages of estrogen receptor-positive cells in young females and in patients with poorly differentiated GC (29–31). The relationship between gender hormones and the prognosis of GC remains controversial. Further studies are needed to determine whether gender affects prognosis in younger patients.

The 5-year overall survival rate for the YG and OG was 59.7 and 65.9%, respectively. Survival in the OG was significantly higher than that in the YG (P=0.049). In previous reports, the prognosis of younger patients was poor and the survival rate was low, particularly in patients with advanced GC (32–34). Delay in diagnosis and the more aggressive biological behavior of GC in younger patients have been suggested as possible causes of poor prognosis (3–5). In our study, we found higher proportions of T4 invasion, peritoneal metastasis, distant metastasis and stage IV in young patients. However, we also found that younger patients have similar outcomes to older patients when matched for tumor stage. Whether the prognosis of GC patients undergoing resection is influenced by age remains unclear, but curative resection is the only chance for long-term survival for GC patients. Some analysis has indicated that younger patients undergoing curative resection have a better prognosis than those who do not undergo the procedure (6–9). Our study also found that patients in the YG who underwent curative resection had a similar 5-year survival rate to patients in the OG who underwent curative resection.

We also compared survival in the YG according to the stage (II–IV) of CG or NCG patients. In our study, there was no benefit regarding survival for all stages between the 2 groups. However, because numerous drugs and regimens have been used in our institution over a period of 30 years, accurate evaluation was difficult. The role of chemotherapy in prolonging life, either with adjuvant or palliative intent, is controversial. There were many prospective randomized trials for adjuvant chemotherapy after curative resection and for unresectable cases. Recently, the large-scale Japanese phase III trial by the Adjuvant Chemotherapy Trial of S-1 for Gastric Cancer (ACTS-GC) group reported the superiority of S-1 as an adjuvant chemotherapy over surgery alone after D2 lymph node dissection (35). Its applicability outside of East Asia is uncertain and the First-Line Advanced Gastric Cancer Study (FLAGS) in advanced disease that compared cisplatin and S-1 vs. cisplatin and fluoropyridines in non-Asian countries was negative (36). Median survival has gradually improved, but is still <1 year and standard treatment remains a matter of debate.

Other studies have suggested various clinicopathological factors that contribute to poorer survival outcomes (2,4,5,7,10,29). In this study, macroscopic type, depth of invasion, peritoneal metastasis, distant metastasis and curative resection were independent factors in younger patients for reduced survival by multivariate analysis. These results suggest that a more aggressive surgical attitude and early diagnosis should be carried out in younger patients with GC to achieve curative resection, which may improve patient outcomes.

In conclusion, this study demonstrated that young patients with GC who undergo curative resection do not have a worse prognosis than older patients. Early diagnosis, particularly in young females, is vital for a successful curative resection and a better prognosis.

## Figures and Tables

**Table I:** Clinicopathological features of the gastric cancer patients.

	Groups		
		
Factors	≤40 year (n=169) n (%)	>40 year (n=3,649) n (%)	P-value^a^
Gender			
Male	79 (46.8)	2,518 (69.0)	<0.0001^a^
Female	90 (53.3)	1,131 (31.0)	
Family history of GC	10 (5.9)	229 (6.28)	0.851
Tumor location			
Upper third	34 (20.1)	790 (21.7)	0.636
Middle third	70 (41.4)	1,047 (28.7)	0.0004^a^
Lower third	40 (23.7)	1,341 (36.8)	0.0005^a^
Whole stomach	23 (13.6)	329 (9.0)	0.044^a^
Gastric stump	2 (1.2)	142 (3.9)	0.071
Esophageal invasion	16 (9.5)	299 (8.2)	0.556
Duodenal invasion	4 (2.4)	141 (3.9)	0.312
Macroscopic type			
Type 0	62 (36.7)	1,685 (46.2)	0.016^a^
Type 1	0 (0)	87 (2.4)	0.042^a^
Type 2	11 (6.5)	431 (11.8)	0.035^a^
Type 3	41 (24.3)	864 (23.7)	0.862
Type 4	40 (23.7)	347 (9.5)	<0.0001^a^
Type 5	15 (8.9)	235 (6.4)	0.211
Histological type			
pap	0 (0)	163 (4.5)	0.008^a^
tub	24 (14.2)	1,861 (51.0)	<0.0001^a^
por	66 (39.1)	943 (25.4)	0.0002^a^
sig	75 (44.4)	600 (16.4)	<0.0001^a^
muc	4 (2.4)	82 (2.2)	0.836
Depth of invasion (T)			
1a	42 (24.9)	910 (24.9)	0.010^a^
1b	18 (10.7)	757 (20.7)	
2	10 (5.9)	242 (6.6)	
3	8 (4.7)	252 (6.9)	
4a	57 (33.7)	1,019 (27.9)	
4b	17 (10.1)	231 (6.3)	
x	17 (10.1)	238 (6.5)	
LN metastasis (N)			
0	96 (56.8)	2,033 (55.7)	0.292
1	11 (6.5)	365 (10.0)	
2	10 (5.9)	337 (9.2)	
3a	20 (11.8)	346 (9.5)	
3b	13 (7.7)	311 (8.5)	
x	19 (11.2)	257 (7.0)	
No. of metastatic LNs	4.0±8.0	4.1±9.0	0.939
Hepatic metastasis (H)	4 (2.4)	203 (5.6)	0.072
Peritoneal metastasis (P)	33 (19.6)	414 (11.5)	0.0014^a^
Distant metastasis (M)	20 (11.8)	341 (9.4)	0.280
Stage			
I	68 (40.2)	1,765 (48.4)	0.019^a^
II	30 (17.6)	471 (12.9)	
III	23 (13.6)	628 (17.2)	
IV	48 (28.4)	782 (21.5)	

a P<0.05, statistical significance.

Tx, Nx: degree is unknown since the primary tumor was not resected. pap, papillary adenocarcinoma; tub, tubular adenocarcinoma; por, poorly differentiated adenocarcinoma; sig, signet ring cell carcinoma; muc, mucinous; GC, gastric cancer; LN, lymph node.

**Table II:** Surgical characteristics of the gastric cancer patients.

	Groups		
		
Factors	≤40 year (n=169) n (%)	>40 year (n=3,649) n (%)	P-value^a^
Operation procedure			
Total	52 (30.8)	936 (25.7)	0.138
Proximal	3 (1.9)	195 (5.3)	0.041^a^
Distal	97 (57.4)	2,116 (58.0)	0.879
Partial resection	0 (0)	164 (4.5)	0.005^a^
Bypass	0 (0)	49 (1.3)	0.130
Open and closure	8 (4.7)	82 (2.3)	0.037^a^
Non-surgery	9 (5.3)	107 (2.9)	0.076
Combined resection	45 (26.6)	789 (21.6)	0.124
Pancreas	21 (12.4)	197 (5.4)	0.0001^a^
Spleen	40 (23.7)	588 (16.1)	0.010^a^
Liver	1 (0.6)	16 (0.4)	0.770
Gallbladder	5 (3.0)	199 (5.5)	0.159
Large intestine	8 (4.7)	37 (1.0)	<0.0001^a^
Ovary	3 (1.8)	4 (0.1)	<0.0001^a^
Diaphragm	2 (1.2)	22 (0.6)	0.351
Others	1 (0.6)	10 (0.3)	0.451
Lymphadenectomy			
D0	3 (2.0)	217 (6.4)	0.0015^a^
D1	30 (19.7)	988 (29.0)	
≥D2	119 (78.3)	2,205 (64.7)	
Gastric resection	152 (89.9)	3,411 (93.5)	0.072
Non-gastric resection	17 (10.1)	238 (6.5)	
Curative resection	112 (73.7)	2,728 (80.0)	0.059
Non-curative resection	40 (26.3)	683 (20.0)	
Chemotherapy	69 (40.8)	1,180 (32.3)	0.021^a^

a P<0.05 indicates statistical significance.

**Table III:** Analyses for prognostic factors for gastric cancer; in young patients.

	Multivariate analysis		
	
Factors	Hazard ratio	95% CI	P-value^a^
Gender			
Male vs. female			0.971
Tumor location			
L, M vs. U			0.917
Macroscopic type			
Type 1–2 vs. type 3–4	8.684	1.451–169.115	0.014^a^
Histological type			
Differ. vs. undiffer			0.814
Depth of invasion			
T1–2 vs. T3–4	3.346	1.054–10.838	0.041^a^
LN metastasis			
N0–1 vs. N2–3			0.175
Hepatic metastasis			
H(−) vs. H(+)			0.083
Peritoneal metastasis			
P(−) vs. P(+)	7.229	2.241–24.323	0.001^a^
Distant metastasis			
M(−) vs. M(+)	4.271	1.284–15.842	0.018^a^
Lymphadenectomy			
D0–1 vs. D2–3			0.736
Curative resection			
No vs. Yes	6.322	2.423–21.255	0.021^a^

a P<0.05 indicates statistical significance.

CI, confidence interval. Determined by the Cox proportional hazard model.
